# Multimodal feature fusion-based graph convolutional networks for Alzheimer’s disease stage classification using F-18 florbetaben brain PET images and clinical indicators

**DOI:** 10.1371/journal.pone.0315809

**Published:** 2024-12-23

**Authors:** Gyu-Bin Lee, Young-Jin Jeong, Do-Young Kang, Hyun-Jin Yun, Min Yoon

**Affiliations:** 1 Department of Nuclear Medicine, Dong-A University College of Medicine and Medical Center, Busan, Korea; 2 Department of Applied Mathematics, Pukyong National University, Busan, Korea; 3 Institute of Convergence Bio-Health, Dong-A University, Busan, Korea; Naval Medical University, CHINA

## Abstract

Alzheimer’s disease (AD), the most prevalent degenerative brain disease associated with dementia, requires early diagnosis to alleviate worsening of symptoms through appropriate management and treatment. Recent studies on AD stage classification are increasingly using multimodal data. However, few studies have applied graph neural networks to multimodal data comprising F-18 florbetaben (FBB) amyloid brain positron emission tomography (PET) images and clinical indicators. The objective of this study was to demonstrate the effectiveness of graph convolutional network (GCN) for AD stage classification using multimodal data, specifically FBB PET images and clinical indicators, collected from Dong-A University Hospital (DAUH) and Alzheimer’s Disease Neuroimaging Initiative (ADNI). The effectiveness of GCN was demonstrated through comparisons with the support vector machine, random forest, and multilayer perceptron across four classification tasks (normal control (NC) vs. AD, NC vs. mild cognitive impairment (MCI), MCI vs. AD, and NC vs. MCI vs. AD). As input, all models received the same combined feature vectors, created by concatenating the PET imaging feature vectors extracted by the 3D dense convolutional network and non-imaging feature vectors consisting of clinical indicators using multimodal feature fusion method. An adjacency matrix for the population graph was constructed using cosine similarity or the Euclidean distance between subjects’ PET imaging feature vectors and/or non-imaging feature vectors. The usage ratio of these different modal data and edge assignment threshold were tuned by setting them as hyperparameters. In this study, GCN-CS-com and GCN-ED-com were the GCN models that received the adjacency matrix constructed using cosine similarity (CS) and the Euclidean distance (ED) between the subjects’ PET imaging feature vectors and non-imaging feature vectors, respectively. In modified nested cross validation, GCN-CS-com and GCN-ED-com respectively achieved average test accuracies of 98.40%, 94.58%, 94.01%, 82.63% and 99.68%, 93.82%, 93.88%, 90.43% for the four aforementioned classification tasks using DAUH dataset, outperforming the other models. Furthermore, GCN-CS-com and GCN-ED-com respectively achieved average test accuracies of 76.16% and 90.11% for NC vs. MCI vs. AD classification using ADNI dataset, outperforming the other models. These results demonstrate that GCN could be an effective model for AD stage classification using multimodal data.

## 1 Introduction

Alzheimer’s disease (AD), the most common type of dementia, is a neurodegenerative disorder that starts with mild memory and cognitive impairments and can progress to severe brain damage, impairing physical abilities and daily functioning [1–5]. Since there is currently no perfect cure for AD, early diagnosis is important for timely intervention and treatment planning, to slow progression of symptoms and enhance the quality of life [1–5]. Mild cognitive impairment (MCI), a stage between normal control (NC) and dementia, also poses significant risk of progression, necessitating early diagnosis [6]. A comprehensive review [6] reported that approximately 20–40% of MCI cases progress to dementia, with an annual progression rate of approximately 10–15%.

Multimodal data comprise comprehensive information from various sources that cannot be obtained using a single modality [7, 8]. In prediction tasks, the objective of multimodal models is to accurately predict unseen data by effectively integrating and learning information across multiple modalities. In practice, doctors clinically diagnose AD stage by evaluating various types of information from several modalities for a subject. Accordingly, AD stage classification studies using multimodal data have been increasing recently and demonstrating high performance [9–15]. Among these, Zhang et al. [15] proposed a multimodal graph neural network (GNN) that leverages structural magnetic resonance imaging (sMRI), F-18 Fluorodeoxyglucose brain positron emission tomography (FDG PET) scans, and phenotypic information for AD stage classification. Their model achieved an average accuracy of 96.68% for NC vs. AD classification and 78.00% for the stable MCI vs. progressive MCI classification.

Research has been ongoing on the 3D convolutional neural network (CNN) for extracting meaningful spatial features from 3D medical images [5, 16–19]. Recent reviews have also emphasized the role of explainable artificial intelligence (XAI) models, including CNNs, in improving transparency and trust in AD stage classification systems [20]. Since 3D medical images consist of a large number of voxels, a 3D CNN requires more data to extract meaningful features than when learning 2D image data. To address this problem, several techniques such as data augmentation, transfer learning, and pretrained models have been proposed; however, they are not always effective for AD stage classification [21]. To fundamentally address this problem, an efficiently learnable CNN model is required. A dense convolutional network (DenseNet) was designed to efficiently learn image data using a dense connectivity pattern, which substantially reduces the number of parameters through feature reuse [22]. Wang et al. [17] proposed an ensemble based-3D DenseNet model, using T1-weighted MRI for AD stage classification. This model achieved an accuracy of 97.19% in NC vs. MCI vs. AD classification.

The graph neural network belongs to a category of artificial neural networks that analyze graph-structured data consisting of nodes and edges [23–25]. A node can represent an entire observation or one or more features of the observation. An edge signifies the particular pairwise relationship between nodes. GNNs capture complex patterns within a graph using message passing and aggregation mechanisms to update node and graph embeddings, making them suitable for specific tasks [23–25]. Among the various GNN models, the graph convolutional network (GCN) generalizes the convolution operation of a CNN, which is suited for regular Euclidean data such as 2D images, to irregular non-Euclidean data [23–26]. Thus, the GCN can simultaneously learn both image and non-image data and their interactions if the graph data includes both types. The GCN receives both a node feature matrix and an adjacency matrix as input. The node feature matrix is constructed by stacking node feature vectors. The adjacency matrix is constructed to describe the connectivity between nodes as a matrix, where each element indicates the presence or absence of an edge and its weight, if it exists.

In AD stage classification studies, graph data are primarily constructed in two ways. For graph-level classification in brain network graph analysis [27–29], brain regions are represented as nodes, with edges indicating the structural or functional connections that exist between these regions. For node-level classification in population graph analysis [14, 15, 30–32], individual subjects are represented as nodes, with edges indicating pairwise similarities between subjects. Kazi et al. [31] employed a multimodal GCN for node-level AD stage classification using diverse biomarkers (MR, PET imaging, cognitive tests, cerebrospinal fluid (CSF) biomarkers, etc.) as node features and the apolipoprotein E (ApoE) genotype, FDG PET imaging, age, and gender for edge assignment. In their approach, each GCN receives distinct adjacency matrices constructed using each of the four features and the same node feature matrix. The final prediction is made by applying a self-attention mechanism to the logits of each GCN. This method achieved an accuracy of approximately 76% in NC vs. MCI vs. AD classification. Lin et al. [14] proposed a framework based on both 3D DenseNet and GCN for node-level AD stage classification using sMRI, demographic information, and neuropsychological tests. Their study primarily focused on the effect of edge assignment on the performance of GCN. The node feature matrix is constructed by extracting imaging feature vectors from 3D sMRI images using 3D DenseNet as the feature extractor. The adjacency matrix is constructed by assigning edges based on the similarity between the subjects’ imaging feature vectors and/or non-imaging features. Their multimodal GCN, based on the adjacency matrix constructed solely using the non-imaging feature clinical dementia rating scale sum of boxes (CDR-SB), achieved an accuracy of 89.4% in the NC vs. MCI vs. AD classification.

In this study, we noted that the best performance of the GCN [14] was observed when the edges were assigned using only the non-imaging feature CDR-SB. Additionally, the study excluded non-imaging features from the node feature matrix and did not conduct cross validation (CV). The incomplete use of multimodal data and the absence of CV served as the initial motivation for our study. We expected that the best performance of multimodal GCN would be achieved when image and non-image data were used for edge assignment. The objective of our study was to demonstrate that the GCN could be an effective model for AD stage classification using multimodal data.

To achieve this objective, we employed the GCN for node-level AD stage classification using F-18 florbetaben (FBB) PET images and clinical indicators collected from Dong-A University Hospital (DAUH) and Alzheimer’s Disease Neuroimaging Initiative dataset (ADNI). 3D DenseNet was utilized as a feature extractor to obtain PET imaging feature vectors from the 3D FBB PET images [14]. In the population graph, node feature vectors of each subject were the combined feature vectors concatenating the PET imaging feature vectors and non-imaging feature vectors consisting of clinical indicators through a multimodal feature fusion method [7, 8]. Edges were assigned based on either cosine similarity or the Euclidean distance between the subjects’ PET imaging feature vectors and/or non-imaging feature vectors. Additionally, by setting hyperparameters during population graph construction, the usage ratio between PET imaging features and clinical indicators, as well as the threshold for edge assignment were tuned. To achieve reliable results, GCN was compared with support vector machine (SVM), random forest (RF), and multilayer perceptron (MLP), using a modified nested CV (stratified nested 5 × 4-fold CV described in Section 2.6) across four classification tasks (NC vs. AD, NC vs. MCI, MCI vs. AD, and NC vs. MCI vs. AD), with all these models receiving the same combined feature vectors as input.

The population graph construction method and the use of the modified nested CV are key contributions that distinguish our study. Previous studies on AD stage classification using FBB PET images [5, 21, 33, 34] faced challenges in classifying MCI from NC and AD, which motivated us to use GCN. The modified nested CV results indicated that GCN outperforms DenseNet, SVM, RF, and MLP models in four AD stage classifications. To the best of our knowledge, this is the first study to apply GCN to multimodal datasets consisting of FBB PET images and clinical indicators.

## 2 Materials and methods

### 2.1 Data acquisition

This study used two multimodal datasets from the DAUH and ADNI. The DAUH multimodal dataset consisted of subjects who underwent their initial FBB brain PET scans between November 6, 2015, and March 6, 2023, and were diagnosed with NC, MCI, or AD by neurologists at DAUH. A total of 468 subjects, with clinical indicators including the mini-mental state examination (MMSE), CDR-SB, global deterioration scale (GDS), and the short version of the geriatric depression scale (SGDepS), were selected. The clinical characteristics of the DAUH subjects were illustrated in Table 1. The labels of *β*-Amyloid(A*β*) positivity, a hallmark of AD characterized by substantial amyloid plaque accumulation in amyloid brain PET images, were determined by a nuclear medicine specialist at DAUH. The DAUH multimodal dataset used for AD stage classification consisted of FBB PET images and six clinical indicators: age, years of education, MMSE, CDR-SB, GDS, and SGDepS.

**Table 1:** Clinical characteristics of the DAUH subjects.

Category	Group			*p*-value			
	NC	MCI	AD	NC vs. MCI vs. AD	NC vs. AD	NC vs. MCI	MCI vs. AD
Number	76	155	237	-	-	-	-
A*β* positivity (N/P)	60/16	90/65	39/198	**<0.001**	<0.001	0.002	<0.001
Gender (Male/Female)	24/52	62/93	114/123	0.028	0.016	0.271	0.140
ApoE4 (0/1/2)	41/12/0	90/41/9	102/94/22	<0.001	<0.001	0.082	0.005
Age	69.34±7.11	68.50±8.74	69.83±9.16	0.353	0.626	0.437	0.148
Education (year)	9.12±4.23	9.8±4.13	9.99±4.36	0.305	0.124	0.253	0.657
MMSE	27.65±1.72	25.11±2.99	19.28±4.23	<0.001	<0.001	<0.001	<0.001
CDR-SB	0.83±0.63	1.49±0.70	4.72±2.65	<0.001	<0.001	<0.001	<0.001
GDS	2.03±0.47	3.01±0.27	4.20±0.67	<0.001	<0.001	<0.001	<0.001
SGDepS	5.01±4.54	4.94±3.84	6.24±4.94	0.009	0.045	0.906	0.003
CDR	0.42±0.18	0.50±0.00	0.79±0.45	<0.001	<0.001	<0.001	<0.001

Statistics for the numerical variables are reported as mean ± standard deviation (SD)

Abbreviation: A*β*: *β*-Amyloid; N: Negative; P: Positive; ApoE4: the number of ApoE E4 alleles; CDR: Clinical Dementia Rating scale.

For the calculation of *p*-values, Welch’s t-test was conducted on numerical variables following Welch’s one-way ANOVA. However, for CDR, due to the variance of the MCI case being zero, a one-way ANOVA was performed instead. For categorical variables, *p*-values were calculated by conducting a chi-square test.

The ADNI multimodal dataset consisted of subjects who underwent their initial FBB brain PET scans and were diagnosed with NC, MCI, or AD. A total of 88 subjects with clinical indicators, including the MMSE, CDR-SB, total score of geriatric depression scale (GDTOTAL), and total score of functional activities questionnaire (FAQTOTAL), were selected for external validation through the Analysis Ready Cohort (ARC) Builder (https://ida.loni.usc.edu/explore/jsp/search/search.jsp?project=ADNI). The clinical characteristics of the ADNI subjects were illustrated in Table 2. Note that the methodologies described in this chapter are primarily based on the DAUH multimodal dataset. The external validation using the ADNI multimodal dataset is described in detail in Section 3.8.

**Table 2:** Clinical characteristics of the ADNI subjects.

Category	Group			*p*-value			
	NC	MCI	AD	NC vs. MCI vs. AD	NC vs. AD	NC vs. MCI	MCI vs. AD
Number	30	30	28	-	-	-	-
Age	70.04±1.33	71.47±2.16	75.60±6.83	<0.001	0.010	<0.001	0.013
Gender (male / female)	11 / 19	17 / 13	19 / 9	0.053	0.195	0.034	0.543
MMSE	29.30±0.83	27.70±1.85	23.17±3.56	<0.001	<0.001	<0.001	<0.001
CDR-SB	0.01±0.09	1.71±1.54	5.73±2.39	<0.001	<0.001	<0.001	<0.001
GDTOTAL	0.56±0.97	2.33±2.07	2.92±2.38	<0.001	<0.001	<0.001	0.950
FAQTOTAL	0.06±0.25	2.03±2.53	16±6.79	<0.001	<0.001	<0.001	<0.001

Statistics for the numerical variables are reported as mean ± standard deviation (SD)

For the calculation of *p*-values, Welch’s t-test was conducted on numerical variables following Welch’s one-way ANOVA. For categorical variables, *p*-values were calculated by conducting a chi-square test.

### 2.2 Image acquisition and preprocessing

In this study, PET scans of DAUH multimodal dataset were acquired using a Biograph 40 mCT Flow PET/CT Scanner (Siemens Healthcare, Knoxville, TN, USA), operating at 100 kVP and 228 mA with a rotation time of 0.5 seconds, without the use of an intravenous contrast agent. The skulls were scanned from the apex to the base using Ultra HD-PET (True X-TOF) for 90–110 minutes following the injection of F-18 florbetaben.

**Fig 1:**
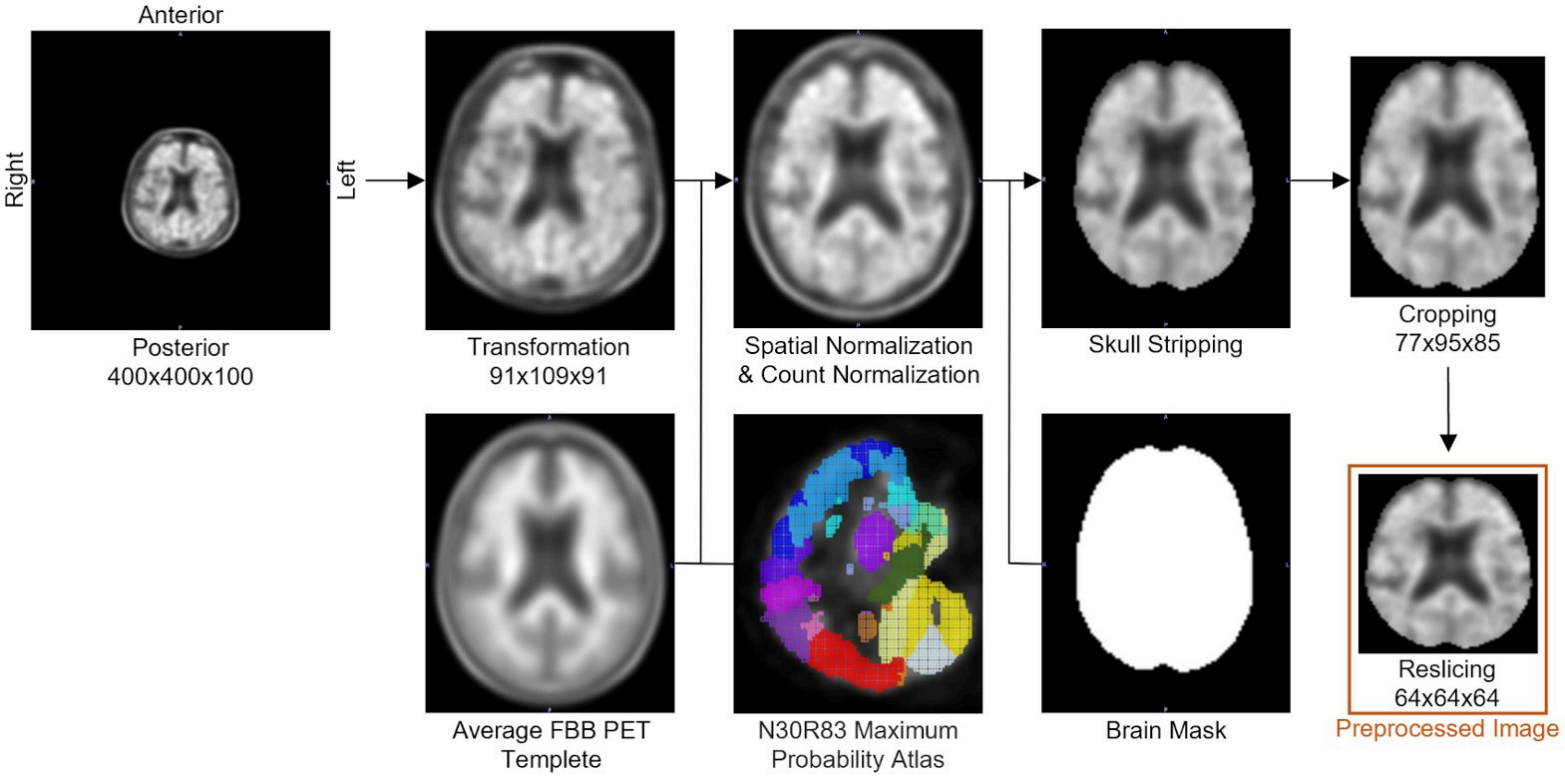
FBB PET image preprocessing pipeline.

For analysis, all PET scans were converted from Digital Imaging and Communications in Medicine (DICOM) to Neuroimaging Informatics Technology Initiative (NIFTI) format using MRIcron. Conventional image preprocessing was performed using the PMOD software (version 4.303, PMOD Technologies Ltd., Zurich, Switzerland) to put the PET images into a form suitable for CNN. The image preprocessing procedure shown in Fig 1 included the following steps.

1. Match: simultaneously loading and aligning a subject’s PET and CT images.
2. Spatial normalization: aligning the matched images with the average FBB PET template.
3. Count normalization: normalizing pixel values against the cerebellum’s average pixel value using the Hammers maximum probability atlas [35].
4. Skull stripping: removing the skull and non-brain regions using a brain mask.
5. Cropping: removing empty space to reduce unnecessary pixels.
6. Reslicing: resizing preprocessed images of size 79 × 95 × 85 to 64 × 64 × 64 using trilinear interpolation in Python.

Due to the dimensional differences between the ADNI and DAUH FBB PET scans, the ADNI FBB PET scans were resliced to match the reference dimensions (91 × 109 × 91) using trilinear interpolation prior to spatial normalization.

### 2.3 3D DenseNet

DenseNet, a deep convolutional neural network architecture proposed by Huang et al. [22], was designed to enhance the flow of information between layers. DenseNet employs a dense connectivity pattern in which each convolutional layer within a dense block receives feature maps from all previous layers, concatenating the feature maps and passing the result to all subsequent convolutional layers.

For efficient downsampling, DenseNet incorporates transition layers between dense blocks. These transition layers perform convolution and pooling. The DenseNet architecture has several advantages such as alleviating the vanishing gradient problem, enhancing the efficiency of feature propagation, encouraging feature reuse, and substantially reducing the number of parameters.

**Fig 2:**
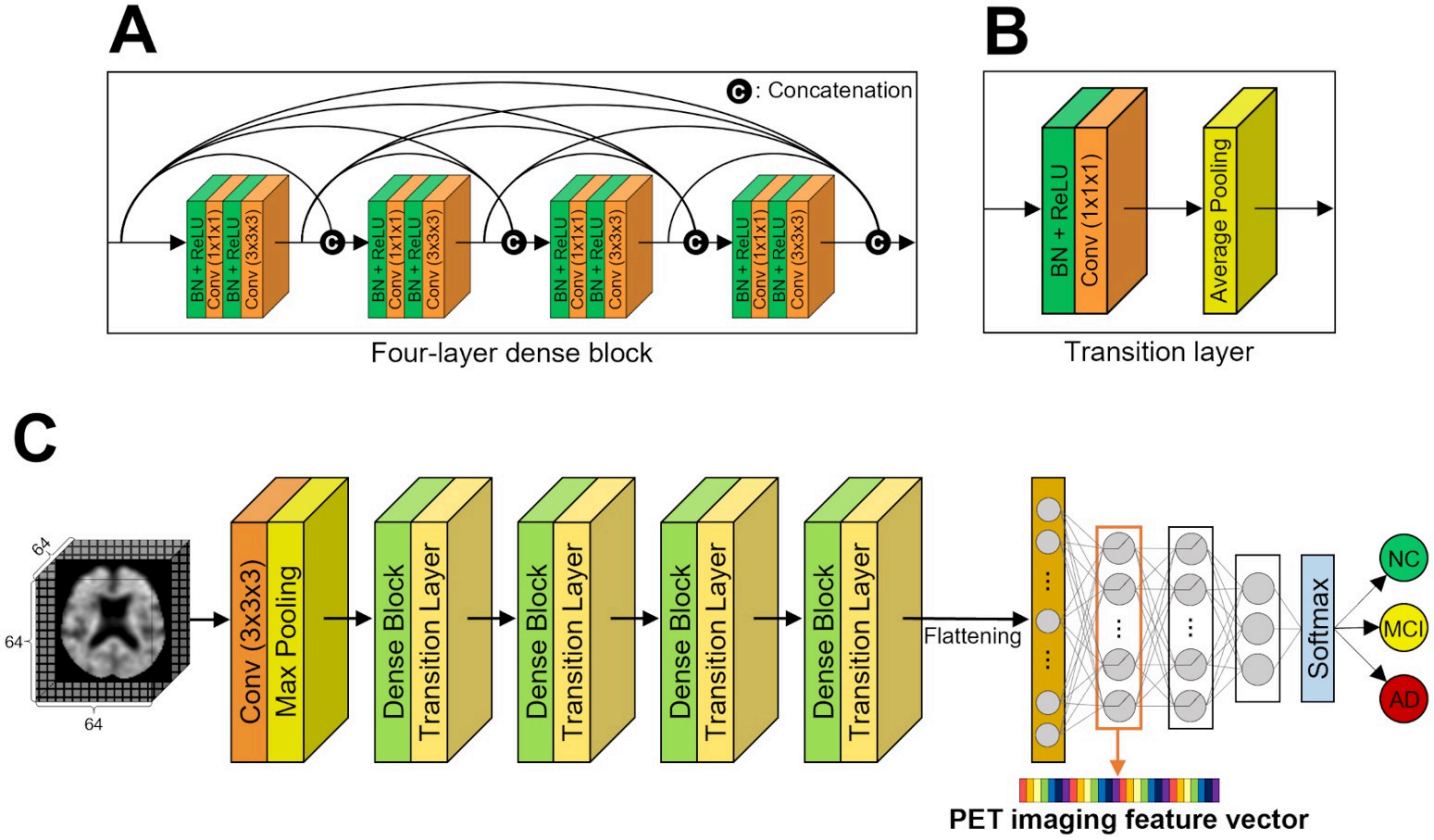
Overall architecture of 3D DenseNet. (A) A four-layer dense block, (B) A transition layer, (C) 3D DenseNet architecture for the multiclass classification.

In this study, we used the DenseNet-BC architecture which additionally incorporates bottleneck layers in the dense blocks and a compression factor *θ*(0 < *θ* < 1) in the transition layers to improve computational efficiency and model compactness [22]. This model was chosen because of its advantages, as the model outperformed the other 3D CNN models in A*β* positivity classification (detailed in S1 Table). Unless otherwise specified, each side of the input was zero-padded by one pixel, and a stride of one was used in all 3 × 3 × 3 convolutions to maintain a fixed feature-map size, whereas a stride of two was used for all the 2 × 2 × 2 pooling for non-overlapping reduction of feature-map size. The overall 3D DenseNet architecture is illustrated in Fig 2.

#### 2.3.1 Dense block

The dense connectivity pattern in a dense block is the core of DenseNet and is utilized for efficient extraction of features from images. The feature-maps of the *l*^*th*^ convolutional layer in a dense block of 3D DenseNet-BC can be formulated as follows:

$$\begin{array}{c} X_{l}=H_{l}([X_{0},X_{1},\ldots ,X_{l-1}]), \end{array}$$ (1)

where, $\left[X_{0},X_{1},\ldots ,X_{l-1}\right]$ denotes the concatenation of the 3D feature-maps produced by all preceding convolutional layers in the dense block; […] refers to the concatenation operation; *H*_*l*_(⋅) is a composite function comprising six consecutive operations: batch normalization (BN), rectified linear unit (ReLU), 1 × 1 × 1 convolution (Conv), BN, ReLU, and 3 × 3 × 3 Conv. Each function *H*_*l*_ produces *k* output feature-maps, with hyperparameter *k* indicating the growth rate. Each 1 × 1 × 1 bottleneck convolutional layer produces *b* × *k* feature-maps, where *b* denotes a hyperparameter. Fig 2A illustrates an example of a four-layer dense block.

#### 2.3.2 Transition layer

The transition layers in 3D DenseNet-BC play a role in reducing the number and size of feature-maps. First, if a transition layer received *m* number of feature-maps from the dense block, the 1 × 1 × 1 convolutional layer in the transition layer produces *θ* × *m* feature-maps after BN-ReLU operations, where 0 < *θ* < 1 refers to the compression factor. In this study, we set *θ* = 0.5, which means the number of feature-maps is halved after passing through the 1 × 1 × 1 convolutional layer because its value is empirically the best in A*β* positivity classification. Second, the size of 0.5 × *m* feature-maps is reduced by 2 × 2 × 2 average pooling. Fig 2B illustrates an example of a general transition layer.

#### 2.3.3 Hpyerparameters of 3D DenseNet

The best hyperparameters of the 3D DenseNet for MCI vs. AD classification, excluding the learning rate and dropout rate, were applied to the remaining three classifications. The reason for this approach was the large number of hyperparameters in DenseNet [18, 23], and the four classification tasks were highly associated. First, the number of convolutions in the initial convolutional layer was set to 2 × *k*, the number of dense blocks to 4, and *θ* to 0.5 [23]. Second, after several empirical hyperparameter experiments, the final search range was set as follows: the number of 3 × 3 × 3 convolutional layers in each of the four dense blocks was either (5, 5, 5, 5) or (3, 6, 12, 8); growth rate *k* was (16, 24, 32); hidden units in the first and second fully connected layers were (128, 256, 512); and (64, 128), respectively. In addition, the batch size was set to 32, the learning rates to 10^−3^ or 10^−4^, and dropout rates to 0.2 or 0.3. Third, the best hyperparameters were determined by conducting a grid search method within the modified nested CV, identifying those that showed the best performance on the validation datasets (detailed in Section 2.6). The best hyperparameters in the MCI vs. AD classification were identified as follows: number of 3 × 3 × 3 convolutional layers in each of the four dense blocks was (3, 6, 12, 8); growth rate *k* was 32; the hidden units in the first and second fully connected layers were 256 and 64, respectively; learning rate was 10^−4^, and dropout rate was 0.2.

Fig 2C illustrates the 3D DenseNet for multiclass classification. The trained 3D DenseNet was employed as a feature extractor to obtain PET imaging feature vectors from 3D FBB PET images, which were then input into the multimodal models. PET imaging feature vectors comprising 256 values were extracted from the first fully-connected layer [14]. To ensure consistency and prevent data leakage, identical seed value was used for all data splits in the modeling process.

### 2.4 Population graph construction

In this study, population graphs were constructed by representing individual subjects as nodes, with edges connecting them based on the similarity of the imaging and/or non-imaging features. In detail, we constructed the undirected graph G(V,E) consisting of a set of nodes $V=\{v_{1},v_{2},\ldots ,v_{N}\}$ and a set of edges E⊆V×V representing the set of connections between nodes, where *N* is the total number of nodes in the graph. Each node *v*_*i*_ represents a subject and possesses a combined feature vector $x_{i}=[x_{i}^{\text{img}},\;x_{i}^{\text{nimg}}]\in R^{262}$, where $x_{i}^{\text{img}}\in R^{256}$ is the PET imaging feature vector extracted through 3D DenseNet, and $x_{i}^{\text{nimg}}\in R^{6}$ is the non-imaging feature vector consisting of age, years of education, MMSE, CDR-SB, GDS, and SGDepS.

The input to the GCN consists of the node feature matrix **X** and adjacency matrix **A**. The node feature matrix $X\in R^{N\times 262}$ consists of the stacked node feature vectors **x**_1_, **x**_2_, …, **x**_*N*_. The adjacency matrix $A\in R^{N\times N}$, which is determined by the set of edges E, represents the pairwise connection information between the nodes. *A*_*ij*_, the element in row *i* and column *j* of **A**, indicates the connection information between *v*_*i*_ and *v*_*j*_. The performance of GCN is significantly affected by the construction of the adjacency matrix [14, 15, 30, 31].

In this study, we used either cosine similarity or the Euclidean distance between the subjects’ imaging feature vectors and/or non-imaging feature vectors to construct the adjacency matrix. The reason for using these two measures was that they are intuitive, and the method for quantifying vector similarity is simple. To ensure that each measure was not affected by the scale of the features, the combined feature vectors were standardized using the mean and SD of the training dataset before edge assignment.

1. Weighted adjacency matrix based on cosine similarity **A**^CS^
  $$\begin{array}{c} A_{ij}^{\text{CS}}=\{\begin{array}{cc} \beta A_{ij}^{\text{img}}+\left(1-\beta \right)A_{ij}^{\text{nimg}} & \text{if}\;\beta A_{ij}^{\text{img}}+\left(1-\beta \right)A_{ij}^{\text{nimg}}\geq \alpha_{\text{CS}}\\ 0 & \text{otherwise} \end{array} \end{array}$$ (2)
  $$\begin{array}{c} A_{ij}^{\text{img}}=\frac{z_{i}^{\text{img}}\cdot z_{j}^{\text{img}}}{∥z_{i}^{\text{img}}∥∥z_{j}^{\text{img}}∥},\;A_{ij}^{\text{nimg}}=\frac{z_{i}^{\text{nimg}}\cdot z_{j}^{\text{nimg}}}{∥z_{i}^{\text{nimg}}∥∥z_{j}^{\text{nimg}}∥} \end{array}$$ (3)
  where the **A**^img^, **A**^nimg^, and **A**^CS^ denote the weighted adjacency matrices based on cosine similarity, which were constructed using standardized PET imaging feature vectors $z_{i}^{\text{img}}$, standardized non-imaging feature vectors $z_{i}^{\text{nimg}}$, and a combination of both, respectively. The *α*_CS_ and *β* are the hyperparameters denoting the cosine similarity threshold and the usage ratio between **A**^img^ and **A**^nimg^ in constructing **A**^CS^, respectively. Initially, the values for *α*_CS_ in the range of 0 to 1 and *β* from 0 to 1, were explored in intervals of 0.1. Subsequently, a more detailed search was conducted at 0.05 intervals around the values that resulted in the best performance of the GCN on the validation dataset.
2. Unweighted adjacency matrix based on Euclidean distance **A**^ED^
  $$\begin{array}{c} A_{ij}^{\text{ED}}=\{\begin{array}{cc} 1 & \text{if}\;\beta A_{ij}^{\text{img}}+\left(1-\beta \right)A_{ij}^{\text{nimg}}\leq q_{\text{ED}}\\ 0 & \text{otherwise} \end{array} \end{array}$$ (4)
  $$\begin{array}{c} A_{ij}^{\text{img}}=\frac{∥z_{i}^{\text{img}}-z_{j}^{\text{img}}∥}{p^{\text{img}}},\;A_{ij}^{\text{nimg}}=\frac{∥z_{i}^{\text{nimg}}-z_{j}^{\text{nimg}}∥}{p^{\text{nimg}}} \end{array}$$ (5)
  where *q*_ED_ denotes the quantile corresponding to hyperparameter *α*_ED_. This quantile is obtained by sorting the upper triangular elements of *β* **A**^img^ + (1 − *β*)**A**^nimg^ in ascending order because the matrix is symmetric. In Eq (5), the *p*^img^ and *p*^nimg^ denote the number of PET imaging features and non-imaging features, respectively. The reason for dividing by the number of features is that the Euclidean distance, unlike the cosine similarity, is affected by the number of features, even after standardization. The search for *β* was conducted in the same manner as for cosine similarity-based edge assignment method. The *α*_ED_ was initially explored from 1 to 49 in increments of 2. If GCN showed better performance when *α*_ED_ increased, we explored larger values by increasing it by 2.

Unlike **A**^CS^, an unweighted adjacency matrix **A**^ED^ was constructed because determining the quantile of each Euclidean distance requires excessive computational resources in assigning edge weights. The reason for exploring *β* was to find an appropriate usage ratio between image and non-image data for edge assignment. The reason for exploring the thresholds *α*_CS_ and *α*_ED_ was to prevent oversmoothing problem, which can occur in the presence of too many unnecessary edges.

**Fig 3:**
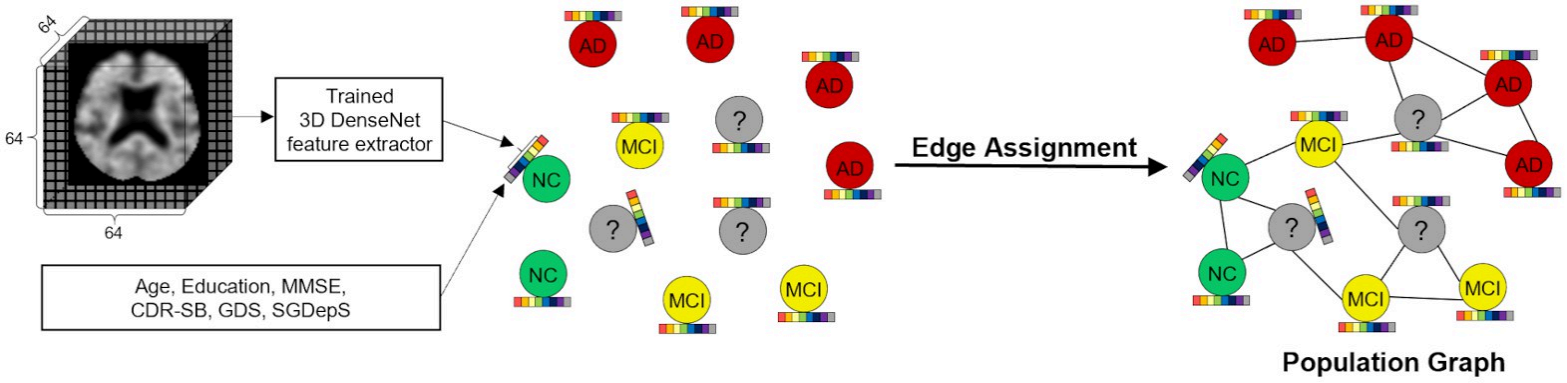
Outline of population graph construction for the multiclass classification.

In summary, a node feature matrix was constructed by employing a multimodal feature fusion method that concatenates the PET imaging feature vectors extracted from 3D DenseNet with non-imaging feature vectors consisting of six clinical indicators. Adjacency matrices were constructed based on either the cosine similarity or Euclidean distance between the standardized PET imaging feature vectors and/or standardized non-imaging feature vectors. Population graph construction is illustrated in Fig 3.

For clarity, the GCN models were categorized according to the input adjacency matrices, with all models receiving the same node feature matrix. Specifically, when *β* is set to 0, GCN-CS-nimg and GCN-ED-nimg are GCN models that respectively receive adjacency matrices **A**^CS^ and **A**^ED^; *β* of 0 indicates that only non-image data are used for edge assignment. Conversely, when *β* is set to 1, GCN-CS-img and GCN-ED-img are GCN models that respectively receive **A**^CS^ and **A**^ED^; *β* of 1 indicates that only PET image data are used for edge assignment. Finally, GCN-CS-com and GCN-ED-com are GCN models that respectively receive **A**^CS^ and **A**^ED^ when *β* ranges from 0.05 to 0.95, indicating the use of both PET image and non-image data for edge assignment.

### 2.5 Graph convolutional networks

**Fig 4:**
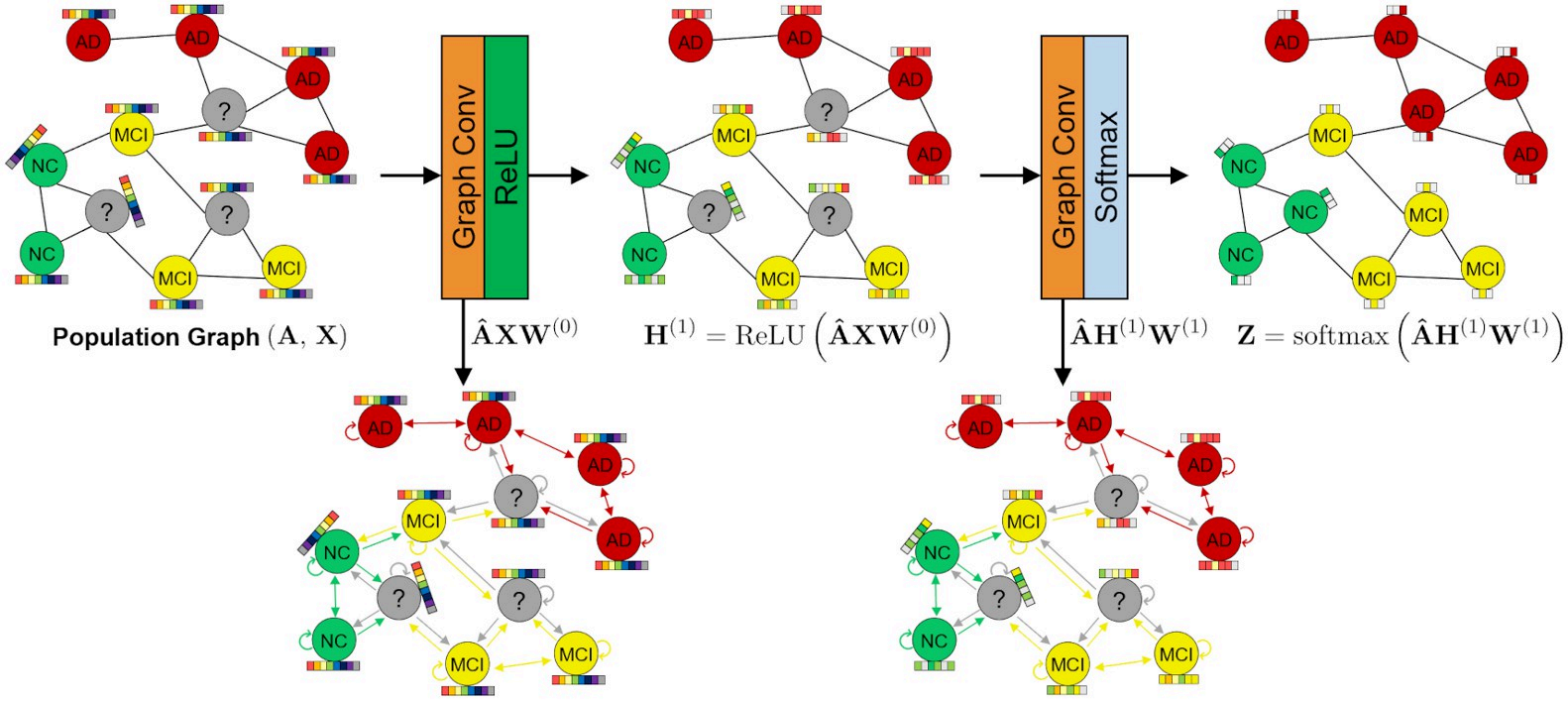
The two-layer GCN architecture for the multiclass classification.

The GCN, a graph neural network architecture proposed by Kipf and Welling [26], has a simple design that effectively learns node representations by aggregating information from neighboring nodes in graph G(V,E), consisting of nodes and edges. This learning process can be formulated using the following layer-wise propagation rule.

$$\begin{array}{c} H^{\left(l+1\right)}=\sigma ({\tilde{D}}^{-\frac{1}{2}}\tilde{A}{\tilde{D}}^{-\frac{1}{2}}H^{\left(l\right)}W^{\left(l\right)}). \end{array}$$ (6)

where the $\tilde{A}=A+I_{N}$ is an adjacency matrix with self-connections added to the undirected graph; $I_{N}\in R^{N\times N}$ is an identity matrix; ${\tilde{D}}_{ii}=\sum_{j}{\tilde{A}}_{ij}$ is a diagonal element of degree matrix $\tilde{D}$, which is a diagonal matrix used for normalization of $\tilde{A}$; and **W**^(*l*)^ is the *l*^*th*^ layer trainable weight matrix. The *σ* is an activation function such as ReLU; **H**^(*l*)^ is the matrix of activations in the *l*^*th*^ layer; **H**^(0)^ = **X**. In this study, a two-layer GCN was employed for semi-supervised node classification on the population graphs constructed in Section 2.4. The output of the two-layer GCN can be formulated as follows:

$$\begin{array}{c} Z=f\left(X,A\right)=\text{softmax}(\hat{A}\text{ReLU}(\hat{A}XW^{\left(0\right)})W^{\left(1\right)}). \end{array}$$ (7)

where the matrix $Z\in R^{N\times C}$ is the output of the two-layer GCN (*C* is the number of classes), and the $\hat{A}={\tilde{D}}^{-1/2}\tilde{A}{\tilde{D}}^{-1/2}$ is the normalized adjacency matrix. Fig 4 illustrates the two-layer GCN for the multiclass classification in this study. The input population graph was constructed by Section 2.4.

The best hyperparameters of the GCN were determined by conducting a grid search method within the modified nested CV, to identify those that showed the best performance on the validation datasets (detailed in Section 2.6). After several empirical hyperparameter experiments, the search range of the GCN was set as follows: the number of hidden units in the graph convolutional layer (64, 128, 256, 512), learning rates (10^−3^, 10^−4^), dropout rates (0.2, 0.3). During GCN modeling, batch gradient descent was used to construct the population graph using the entire dataset.

### 2.6 Hyperparameter tuning and model evaluation method

**Fig 5:**
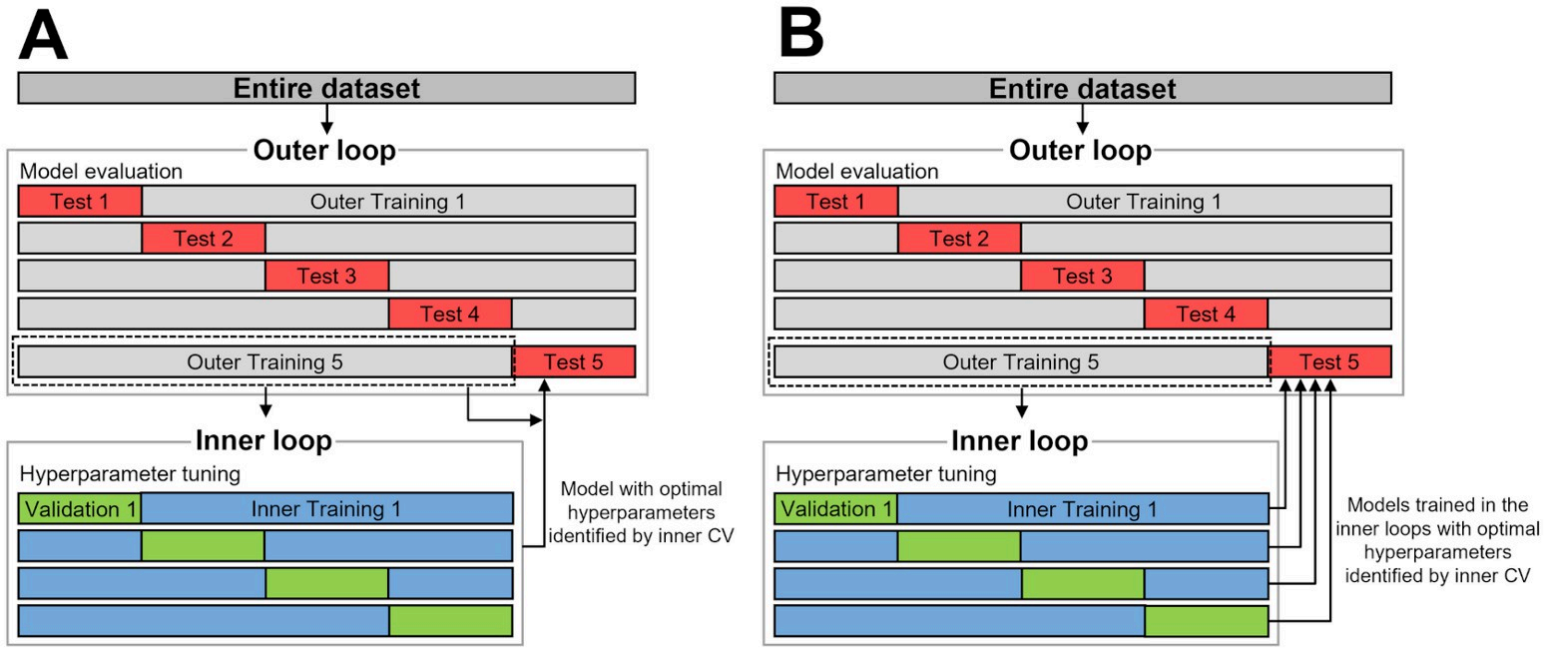
Comparison of nested 5 × 4-fold CV used in this study and traditional nested 5-fold CV. (A) Flowchart of traditional nested 5-fold CV, (B) Flowchart of nested 5 × 4-fold CV used in this study.

To achieve reliable model evaluation and alleviate possible bias caused by random partitioning of the dataset, a stratified nested 5-fold CV was initially considered for both hyperparameter tuning and model evaluation (Fig 5A). However, the number of best epochs for each deep learning model differs according to the hyperparameters and partitioned dataset. To address this challenge and prevent overfitting, an early stopping method was employed as a regularization method in which training is halted if the validation loss does not decrease for a specified number of consecutive epochs.

As the early stopping method requires a validation dataset, the models trained in the inner loops with best hyperparameters, as identified by the inner CV, were used for model evaluation (Fig 5B) instead of training a new model using the outer training dataset with best hyperparameters identified in the inner CV for model evaluation (Fig 5A). Thus, the model evaluation in this study was conducted by averaging the test classification performances of the 20 models in the inner loops. This method uses each of the five folds in the outer loop four times for model evaluation. To differentiate it from the traditional stratified nested 5-fold CV, we refer to it as ‘stratified nested 5 × 4-fold CV’ (referred to as modified nested CV in previous sections) in this study. We believe that this method offers reliable results for model evaluation and comparison. In Fig 5, this method is compared with the traditional nested 5-fold CV.

In detail, all deep learning models were trained for a maximum of 500 epochs using the Adam optimizer with a weight decay of 10^−5^ to prevent overfitting problem. Training was halted when validation loss did not decrease for 20 consecutive epochs. For the loss functions, binary cross-entropy was used for binary classification tasks and categorical cross-entropy for a multiclass classification task. The best hyperparameters were those that showed the lowest average validation loss across all the inner loops. Model evaluation was performed using the 20 models trained in the inner loops with the best hyperparameters.

In binary classification tasks, the model evaluation metrics included accuracy, precision, recall (sensitivity), F1 score, and area under the receiver operating characteristic (ROC) curve (AUC). Accuracy is the ratio of correct predictions to all predictions, precision is the ratio of true positives to positive predictions, and recall is the ratio of true positives to actual positives. The F1 score is the harmonic mean of precision and recall (2 × precision × recall/(precision + recall)). The AUC is the integral of the area under the ROC curve, with values closer to 1 indicating a robust classifier. In a multiclass classification task, the model evaluation metrics include accuracy and the confusion matrix which allows a visualization of the classifier’s predictions for each class. These metrics help comprehensively assess the performance of a classifier.

## 3 Results

### 3.1 Experimental setting

To demonstrate the effectiveness of GCN for AD stage classification using multimodal data, GCN was compared with SVM using the radial basis function (RBF) kernel, RF, and MLP models across four classification tasks using the same combined features vector as those input into the GCN. As RF and SVM do not employ a gradient descent method during learning, we determined the best hyperparameters based on the lowest average validation loss without early stopping in the stratified nested 5 × 4-fold CV. The hyperparameter tuning and model evaluation methods for MLP were the same as those used for GCN. The hyperparameter search ranges for each model are summarized in Table 3. For the MLP models, the number of hidden units in the preceding hidden layer was set to be greater than or equal to the number of hidden units in the subsequent hidden layer. For brevity, SVM using the RBF kernel is referred to as SVM-RBF, and MLPs with one, two, or three hidden layers are referred to as MLP-1HL, MLP-2HL, and MLP-3HL, respectively.

**Table 3:** Hyperparameter search ranges for each model.

Model	Hyperparameter	Search range
SVM-RBF	Regularization parameter	1e-3, 1e-2, …, 1000
	RBF kernel coefficient	1e-3, 1e-2, 1e-1, 1, 2, 3, 4
	Loss function	Hinge loss
RF	Number of trees	100, 200, …, 1000
	Maximum tree depth	10, 20, …, 50
	Minimum samples required to split a node	2, 5
	Minimum samples required in a leaf node	1, 2
	Maximum features considered for node split	sqrt, log_2_
	Loss function	Gini impurity
MLP	Learning rate	1e-5, …, 1e-2
	Dropout rate	0.1, 0.2, 0.3, 0.4, 0.5
	Number of hidden layers	1, 2, 3
	Number of hidden units	64, 128, 256, 512
GCN	Learning rate	1e-5, …, 1e-2
	Dropout rate	0.1, 0.2, 0.3, 0.4, 0.5
	Number of hidden units	64, 128, 256, 512

In all binary classification tasks, recall is considered more important than precision, because it measures the accuracy of identifying subjects with advanced AD stage, whereas precision measures the accuracy of the classifier’s predictions for the advanced AD stage. Therefore, when comparing models with similar accuracies and F1 scores, recall becomes a more critical evaluation metric than precision.

### 3.2 NC versus AD classification performance

In Table 4, we confirmed that the performance of 3D DenseNet, trained only on FBB PET images, did not significantly differ from those of the multimodal models RF, SVM, MLP, GCN-CS-img, and GCN-ED-img. This observation suggests that these multimodal models may not have effectively learned the multimodal data. In particular, GCN-CS-img and GCN-ED-img, which utilized only PET imaging features for edge assignment, underperformed compared to 3D DenseNet. In contrast, the performances of GCN-CS-nimg, GCN-CS-com, GCN-ED-nimg, and GCN-ED-com suggest that they effectively learned multimodal data. Except for the AUC of GCN-ED-com, GCN-CS-com and GCN-ED-com slightly outperformed GCN-CS-nimg and GCN-ED-nimg, respectively. Overall, GCN-ED-com showed the best performance in the NC vs. AD classification.

**Table 4:** Average test performances of each model in NC vs. AD classification (mean ± SD).

Model	Accuracy(%)	Precision(%)	Recall(%)	F1 Score(%)	AUC
3D DenseNet	91.61 ± 3.36	93.25 ± 3.19	95.99 ± 2.73	94.55 ± 2.14	0.9451 ± 0.041
SVM-RBF	91.85 ± 3.07	94.27 ± 2.32	95.04 ± 2.78	94.63 ± 2.05	0.9614 ± 0.029
RF	91.45 ± 2.63	93.46 ± 2.46	95.46 ± 2.84	94.41 ± 1.74	0.9667 ± 0.024
MLP-1HL	92.01 ± 2.98	94.28 ± 2.10	95.25 ± 2.90	94.74 ± 2.01	0.9546 ± 0.033
MLP-2HL	92.33 ± 2.51	94.33 ± 2.37	95.68 ± 2.14	94.97 ± 1.63	0.9585 ± 0.033
MLP-3HL	91.93 ± 3.17	94.76 ± 2.47	94.62 ± 2.94	94.66 ± 2.12	0.9603 ± 0.031
GCN-CS-img	89.93 ± 4.34	93.56 ± 5.29	94.81 ± 2.78	91.77 ± 4.56	0.9320 ± 0.030
GCN-CS-nimg	97.12 ± 1.28	99.25 ± 1.01	96.94 ± 1.02	98.08 ± 0.84	0.9945 ± 0.003
GCN-CS-com	98.40 ± 1.50	99.37 ± 1.31	98.52 ± 1.63	98.93 ± 1.00	0.9970 ± 0.003
GCN-ED-img	90.25 ± 4.11	95.23 ± 2.97	91.76 ± 4.15	93.41 ± 2.88	0.9358 ± 0.047
GCN-ED-nimg	98.24 ± 0.99	98.75 ± 1.01	98.94 ± 1.24	98.84 ± 0.65	**0.9990 ± 0.001**
GCN-ED-com	**99.68 ± 0.63**	**99.79 ± 0.61**	**99.78 ± 0.63**	99.78 ± 0.42	0.9989 ± 0.004

The best performance for each evaluation metric is highlighted in bold.

### 3.3 NC versus MCI classification performance

In Table 5, the average test performance of 3D DenseNet suggests the challenges in classifying NC and MCI using only FBB PET images. The average test recall of 98.22% and precision of 67.41% on 3D DenseNet indicate the tendency to simply predict MCI because MCI cases were approximately twice as numerous as NC cases in the training dataset. Among the multimodal models, GCN-CS-img and GCN-ED-img showed poor performance, possibly because their adjacency matrices were based on cosine similarity and the Euclidean distance between the PET imaging feature vectors extracted by 3D DenseNet, respectively. Similar to the findings for NC vs. AD classification, GCN-CS-com and GCN-ED-com outperformed the other multimodal models. Overall, the GCN-CS-com showed the best performance in NC vs. MCI classification.

**Table 5:** Average test performances of each model in NC vs. MCI classification (mean ± SD).

Model	Accuracy(%)	Precision(%)	Recall(%)	F1 Score(%)	AUC
3D DenseNet	66.88 ± 1.04	67.41 ± 1.17	98.22 ± 3.45	79.90 ± 0.89	0.5233 ± 0.081
SVM-RBF	92.19 ± 3.46	92.87 ± 2.82	95.80 ± 3.83	94.26 ± 2.58	0.9440 ± 0.032
RF	80.21 ± 6.69	81.13 ± 5.20	92.41 ± 8.11	86.15 ± 4.94	0.8480 ± 0.070
MLP-1HL	90.37 ± 3.34	89.98 ± 3.71	96.61 ± 3.14	93.10 ± 2.31	0.9478 ± 0.027
MLP-2HL	90.69 ± 3.87	90.37 ± 3.87	96.61 ± 3.13	93.32 ± 2.67	0.9399 ± 0.031
MLP-3HL	90.04 ± 3.50	90.41 ± 4.95	95.80 ± 4.33	92.84 ± 2.38	0.9426 ± 0.033
GCN-CS-img	66.33 ± 2.80	67.64 ± 1.63	95.80 ± 7.07	79.15 ± 2.61	0.5592 ± 0.090
GCN-CS-nimg	94.57 ± 2.23	95.47 ± 2.65	96.61 ± 2.96	95.98 ± 1.74	0.9646 ± 0.022
GCN-CS-com	94.58 ± 1.88	94.73 ± 2.08	97.41 ± 2.81	96.01 ± 1.42	0.9674 ± 0.021
GCN-ED-img	69.81 ± 9.06	74.07 ± 6.30	85.16 ± 11.19	78.89 ± 7.16	0.7262 ± 0.116
GCN-ED-nimg	92.95 ± 2.99	93.33 ± 3.78	96.61 ± 2.15	94.88 ± 2.08	0.9490 ± 0.025
GCN-ED-com	93.82 ± 2.68	94.08 ± 3.54	97.09 ± 2.25	95.50 ± 1.87	0.9459 ± 0.029

The best performance for each evaluation metric is highlighted in bold.

### 3.4 MCI versus AD classification performance

As shown in Table 6, similar to the previous two binary classification tasks, GCN-CS-com and GCN-ED-com outperformed the other multimodal models, except for precision. Based on these binary classification results, we expect that GCN-ED-com and GCN-CS-com will consistently show the best performance in the multiclass classification task (NC vs. MCI vs. AD).

**Table 6:** Average test performances of each model in MCI vs. AD classification (mean ± SD).

Model	Accuracy(%)	Precision(%)	Recall(%)	F1 Score(%)	AUC
3D DenseNet	84.30 ± 4.21	84.19 ± 6.33	92.31 ± 4.96	87.75 ± 2.71	0.9310 ± 0.038
SVM-RBF	89.42 ± 3.57	91.18 ± 3.36	91.48 ± 4.56	91.25 ± 3.01	0.9553 ± 0.020
RF	87.06 ± 2.80	88.92 ± 3.18	90.00 ± 4.62	89.34 ± 2.41	0.9447 ± 0.020
MLP-1HL	90.69 ± 2.78	92.80 ± 3.21	91.90 ± 4.05	92.26 ± 2.36	0.9637 ± 0.019
MLP-2HL	89.67 ± 3.03	91.64 ± 3.65	91.48 ± 4.65	91.44 ± 2.60	0.9620 ± 0.018
MLP-3HL	90.37 ± 2.83	92.18 ± 2.91	92.01 ± 4.01	92.02 ± 2.40	0.9624 ± 0.017
GCN-CS-img	84.19 ± 5.40	89.65 ± 4.97	83.79 ± 7.38	86.41 ± 4.88	0.9323 ± 0.038
GCN-CS-nimg	92.54 ± 3.30	**96.11 ± 2.28**	91.37 ± 3.89	93.65 ± 2.81	0.9647 ± 0.019
GCN-CS-com	**94.01 ± 2.30**	95.64 ± 1.95	94.42 ± 2.95	**95.00 ± 1.94**	**0.9851 ± 0.010**
GCN-ED-img	85.85 ± 4.39	89.70 ± 3.63	86.64 ± 5.57	88.05 ± 3.85	0.9302 ± 0.035
GCN-ED-nimg	91.97 ± 3.74	95.04 ± 2.27	91.47 ± 4.36	93.20 ± 3.19	0.9622 ± 0.022
GCN-ED-com	93.88 ± 2.88	95.25 ± 2.43	**94.63 ± 3.40**	94.91 ± 2.44	0.9834 ± 0.011

The best performance for each evaluation metric is highlighted in bold.

### 3.5 Multiclass classification performance

In Table 7, the average test accuracy of 3D DenseNet suggests the challenges in classifying NC, MCI, and AD using only FBB PET images. All multimodal models showed better performance than the 3D DenseNet. This result indicates that multimodal data consisting of FBB PET images and clinical indicators can aid multiclass classification. Consistent with our expectations based on the binary classification tasks, GCN-CS-com and GCN-ED-com outperformed the other multimodal models. The average test accuracy of GCN-ED-com was approximately 12.77% higher than that of MLP-3HL, which showed the best performance among the multimodal models, except for GCN. This result indicates that the GCN-ED-com effectively learns multimodal data. GCN-ED-com significantly outperformed GCN-CS-com, unlike the binary classification tasks with similar performances. The reasons for this are discussed in the Discussion Section.

**Table 7:** Average test accuracy of each model in NC vs. MCI vs. AD classification (mean ± SD).

Model	Accuracy(%)
3D DenseNet	71.25 ± 3.16
SVM-RBF	74.83 ± 5.32
RF	73.01 ± 4.50
MLP-1HL	74.99 ± 4.56
MLP-2HL	76.75 ± 4.85
MLP-3HL	77.66 ± 5.16
GCN-CS-img	71.57 ± 3.47
GCN-CS-nimg	82.26 ± 3.04
GCN-CS-com	82.63 ± 4.01
GCN-ED-img	71.52 ± 2.95
GCN-ED-nimg	89.41 ± 2.25
GCN-ED-com	**90.43 ± 1.78**

The best performance is highlighted in bold.

**Fig 6:**
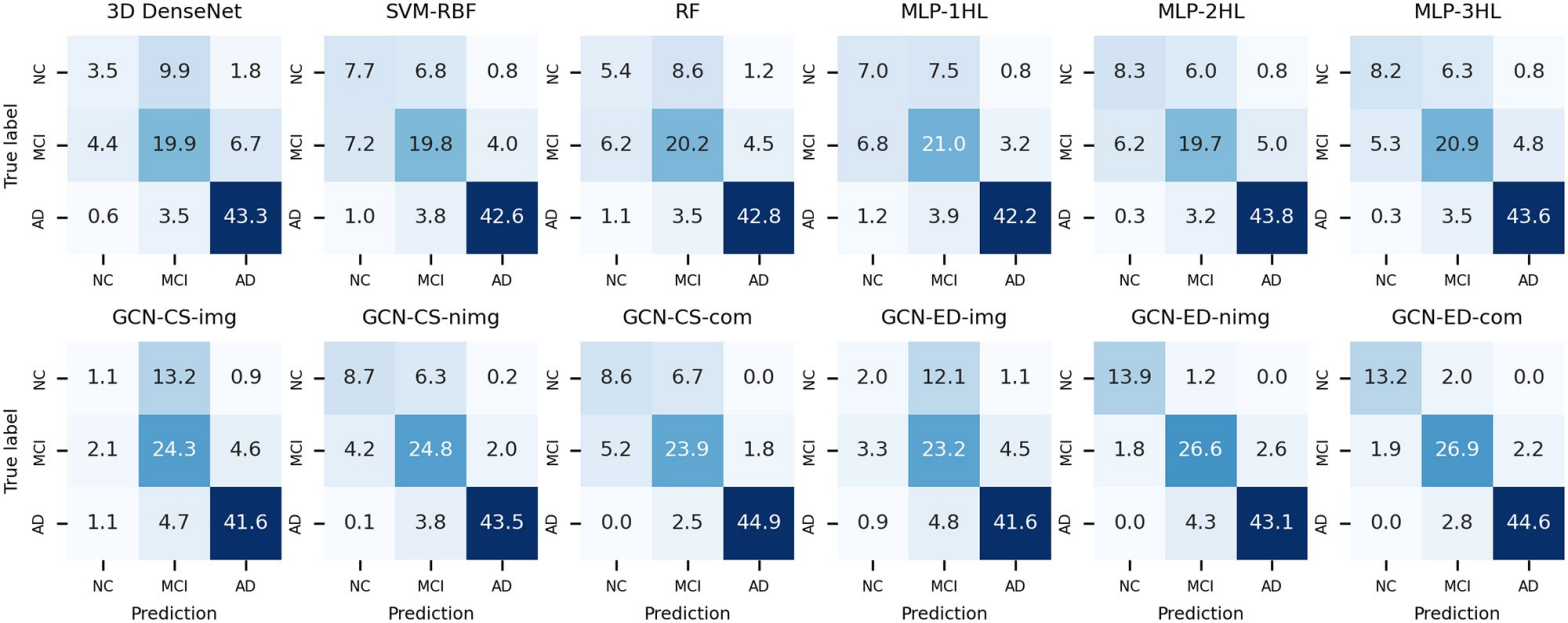
Confusion matrices for each model in the multiclass classification.

Fig 6 illustrates the confusion matrices for each model in multiclass classification. Although GCN-CS-com predicted AD cases slightly more accurately than GCN-ED-com, the latter was notably better at classifying NC and MCI cases. Overall, the GCN-ED-com showed the best performance in multiclass classification. In addition, the robustness test results for the models can be found in S2 Table, where GCN-ED-com also demonstrated the best performance.

### 3.6 Average test accuracy of GCN-CS according to *β*

**Fig 7:**
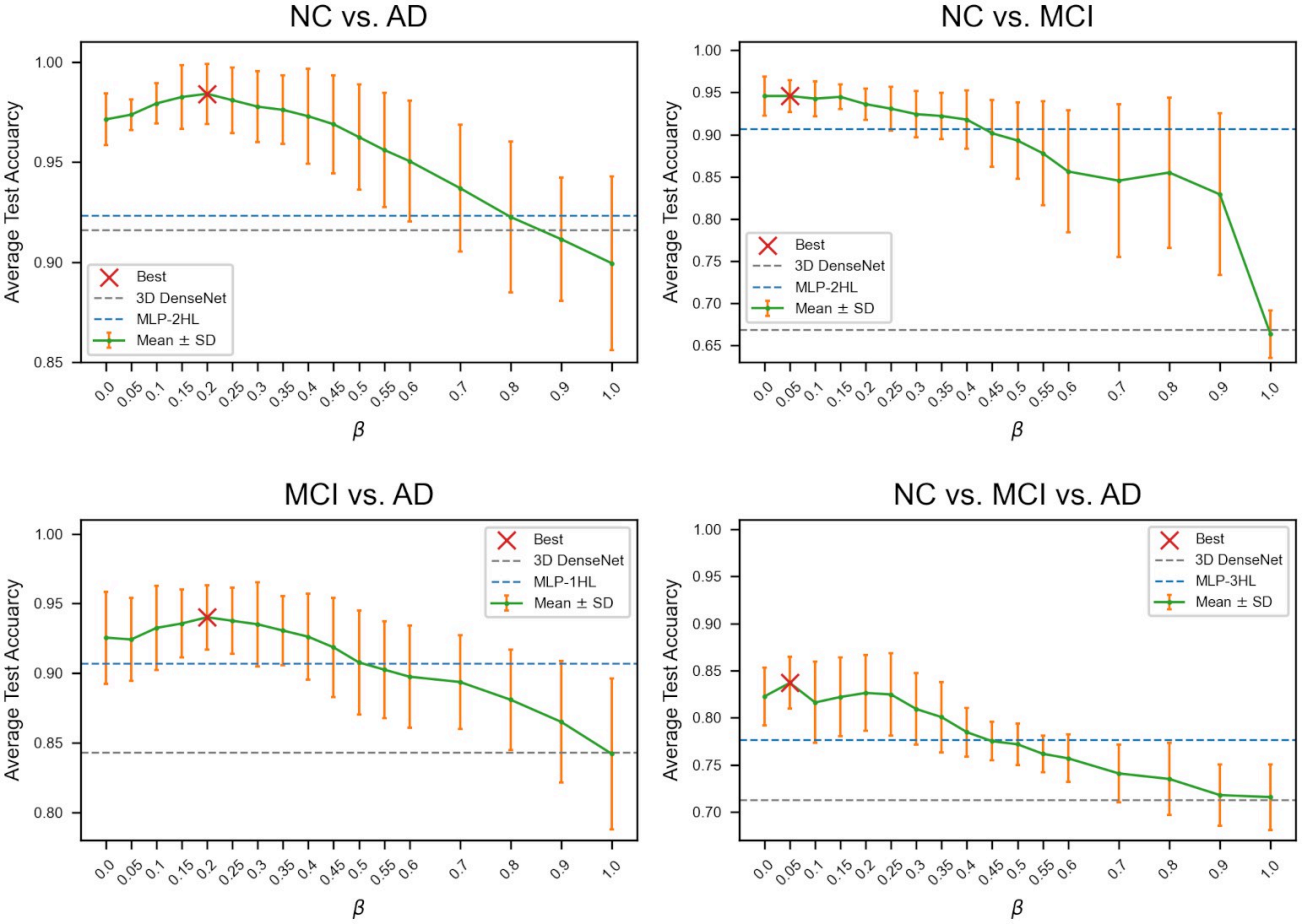
Comparison of average test accuracy of GCN-CS according to *β* in four AD stage classification tasks.

As described in Section 2.4 and Eqs (2) and (3), the hyperparameter *β* denotes the usage ratio between **A**^img^ and **A**^nimg^ in constructing **A**^CS^. In Fig 7, the *β* = 0 and *β* = 1 correspond to GCN-CS-nimg and GCN-CS-img, respectively. For a *β* between 0 and 1, the corresponding model is GCN-CS-com. In NC vs. AD and MCI vs. AD classification, the best performance of GCN-CS-com was observed at *β* = 0.2. In NC vs. MCI and multiclass classification, the best performance of GCN-CS-com was observed at *β* = 0.05. While the cosine similarity thresholds *α*_CS_ were 0.25, 0.55, 0.6, and 0.05 in four AD stage classification, respectively. In all four classification tasks, after achieving the highest accuracy, the average test accuracy tends to decrease as *β* increases. These findings indicate that the clinical indicators are more important than the PET imaging features in cosine similarity-based edge assignment method.

### 3.7 Average test accuracy of GCN-ED according to *β*

**Fig 8:**
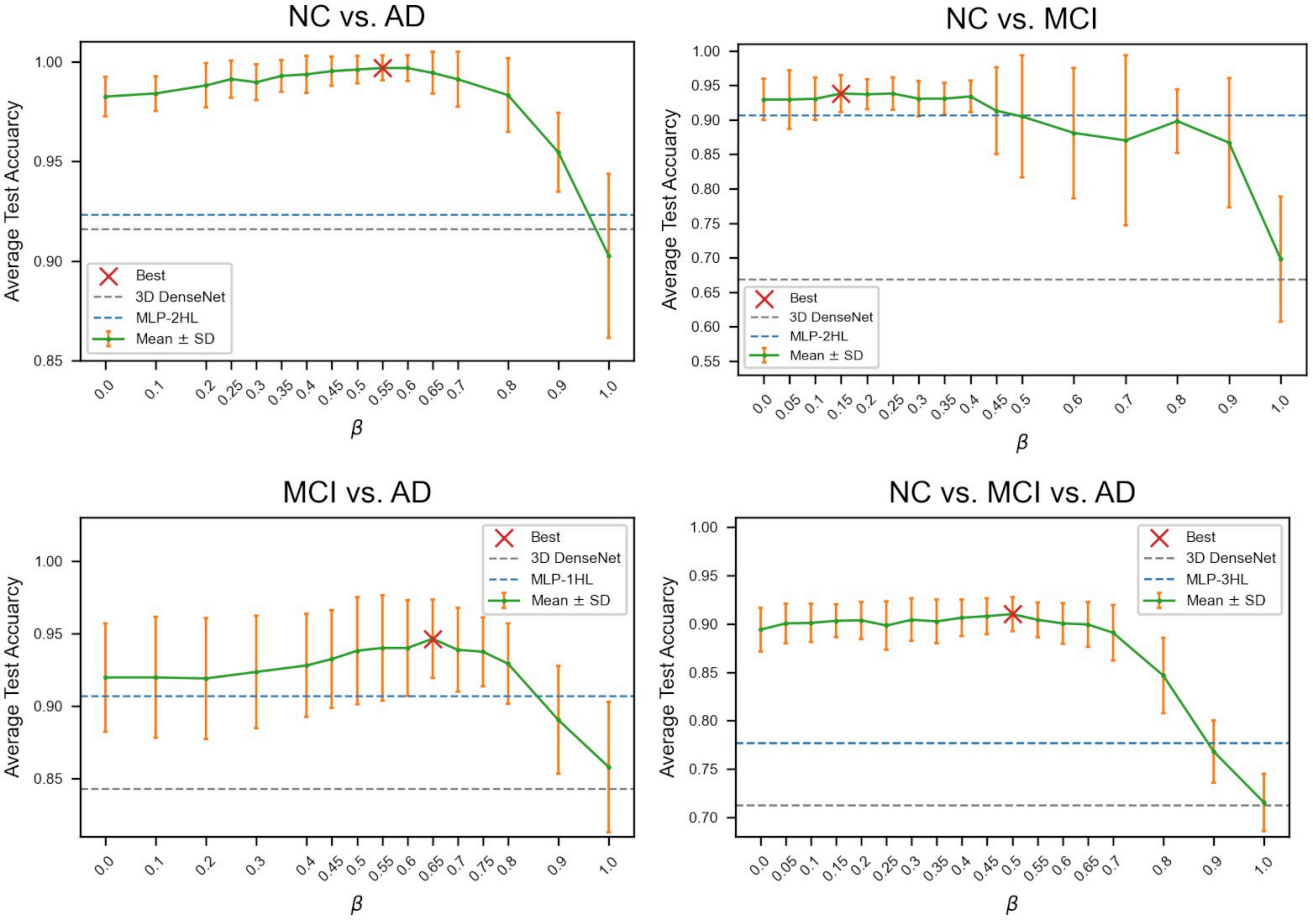
Comparison of average test accuracy of GCN-ED according to *β* in four AD stage classification tasks.

As described in Section 2.4 and Eqs (4) and (5), the hyperparameter *β* denotes the usage ratio between **A**^img^ and **A**^nimg^ in cunstructing **A**^ED^. In Fig 8, in all four classification tasks, after achieving the highest accuracy of GCN-ED, the average test accuracy tends to decrease as *β* increases similar to Fig 7. While the Euclidian distance quantile thresholds *α*_ED_ were 31, 15, 47, and 39 in four AD stage classification, respectively. These findings indicate that the clinical indicators are more important than PET imaging features in the Euclidean distance-based edge assignment method.

### 3.8 External validation

To further validate the effectiveness of the GCN in AD stage classification, we conducted an external validation using the ADNI multimodal dataset for NC vs MCI vs AD classification. Details of the ADNI dataset can be found in Table 2. The differences in the modeling approach used for the DAUH and ADNI datasets are the size of the 3D DenseNet and the hyperparameter search range for the MLP and GCN models. Specifically, considering the smaller number of ADNI subjects, the growth rate of the 3D DenseNet was reduced to 12, and a fully connected layer with 32 hidden units was included to downsize the 3D DenseNet. The candidate hidden units for the MLP were set to 32, 64, 128, and 256, while the hidden units for the GCN were set to 32, and dropout was not applied.

As shown in Table 8, the average test accuracy of the 3D DenseNet indicates the challenges in NC vs. MCI vs. AD classification. This may be due to the limited number of subjects, differences in PET manufacturers and settings, and the lower quality of some FBB PET images. However, the lower performance of the 3D DenseNet is a separate issue, as the primary objective of this study is to demonstrate the effectiveness of the GCN in AD stage classification using multimodal data. The average test accuracy of the GCN-ED-com outperformed the other models, as it did with the DAUH dataset. In addition, the robustness test results for the models can be found in S3 Table, where GCN-ED-com also demonstrated the best performance. Thus, the external validation results further support the effectiveness of the GCN in AD stage classification using multimodal data.

**Table 8:** Result of external validation using ADNI multimodal dataset in NC vs. MCI vs. AD classification (mean ± SD).

Model	Accuracy(%)
3D DenseNet	40.94 ± 9.02
RF	79.08 ± 8.04
SVM-RBF	77.43 ± 12.22
MLP-1HL	74.60 ± 9.88
MLP-2HL	75.37 ± 8.59
MLP-3HL	74.28 ± 8.92
GCN-CS-img	72.05 ± 10.34
GCN-CS-nimg	75.32 ± 5.54
GCN-CS-all	76.16 ± 6.75
GCN-ED-img	64.33 ± 14.01
GCN-ED-nimg	85.81 ± 9.92
GCN-ED-com	**90.11 ± 5.28**

The best performance is highlighted in bold.

## 4 Discussion

Our objective was to demonstrate that GCN could be an effective model for AD stage classification using multimodal data consisting of both FBB PET images and clinical indicators collected from the DAUH. This was demonstrated by comparing GCN with SVM, RF, and MLP in the three binary classification tasks and multiclass classification task, using the stratified nested 5 × 4-fold CV. In all the binary classification tasks, GCN-CS-com and GCN-ED-com, which utilized both FBB PET images and clinical indicators for edge assignment, consistently outperformed the other multimodal models. In the multiclass classification task, GCN-ED-com achieved an average test accuracy of 90.43%, significantly outperforming the other models. However, the performances of GCN-CS-img and GCN-ED-img also indicate that GCN is not always an effective model. These results support our initial expectation that leveraging both image and non-image data for edge assignment is the most effective method for population graph construction.

**Fig 9:**
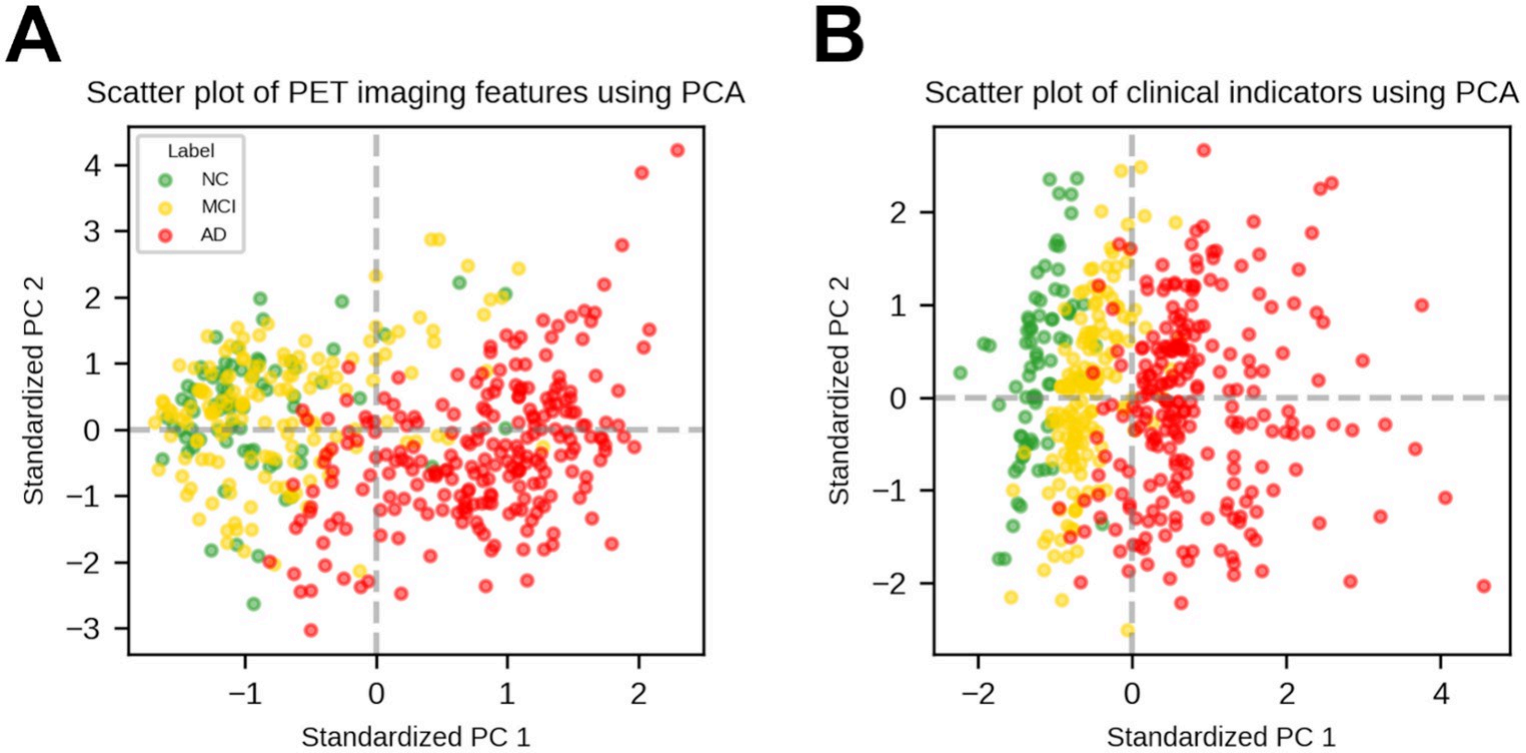
Visualization of PET imaging features and clinical indicators using PCA. (A) Scatter plot of PET imaging features using PCA, (B) Scatter plot of clinical indicators using PCA.

In multiclass classification, unlike binary classifications, the notable performance difference according to the edge assignment method is probably due to the problem with cosine similarity-based edge assignment method. As detailed in Section 2.4, the PET imaging feature vectors and non-imaging feature vectors were standardized using the training dataset before edge assignment. To explore this problem visually, we conducted a principal component analysis (PCA) on standardized PET imaging feature vectors and on standardized non-imaging feature vectors, as illustrated in Fig 9. Fig 9A and 9B illustrate the first two principal components (PCs), which account for approximately 78.47% and 64.79% of the total variance in the standardized PET imaging feature vectors and non-imaging feature vectors, respectively. This allows the visualization of the original high-dimensional dataset, albeit with some loss of information.

Fig 9A indicates that classifying NC and MCI using only PET imaging features is challenging, pointing to the difficulty of properly assigning edges using cosine similarity or the Euclidean distance-based edge assignment method. Fig 9B suggests that the Euclidean distance-based edge assignment method is likely to connect nodes with the same labels. However, using the cosine similarity-based edge assignment method can lead to multiple connections between NC and MCI because cosine similarity is determined by the angle between two vectors [36]. This is indirectly confirmed by the confusion matrices of both GCN-CS-nimg and GCN-CS-com in Fig 6, which show poorer classification performances between NC and MCI compared with both GCN-ED-nimg and GCN-ED-com.

Additionally, we numerically analyzed the edges of the input population graphs for GCN-CS-com and GCN-ED-com in NC vs. MCI vs. AD classification, as shown in Table 7. Table 9 presents the average number of edges between labels across 20 population graphs for each similarity measure, represented as ‘number of edges/total possible edges (average percentage ± SD)’. The total possible number of edges is calculated as n+(n2) for the same label and as *n*_1_ × *n*_2_ for different labels. Since the DAUH multimodal dataset includes 76 NC, 155 MCI, and 237 AD subjects, the corresponding edge counts are shown in the Table 9.

**Table 9:** Numerical edge analysis of population graphs according to similarity measure.

Similarity measure	NC-NC	NC-MCI	NC-AD	MCI-MCI	MCI-AD	AD-AD
**Cosine similarity**	2869.20/2926	9982.40/11780	2187.90/18012	8930.20/12090	7987.45/36735	19513.25/28203
	(98.06±0.34%)	(84.74±0.89%)	(12.15±0.92%)	(73.86±1.66%)	(21.74±1.40%)	(69.19±1.89%)
**Euclidean distance**	2237.70/2926	7560.60/11780	1455.75/18012	8929.75/12090	10427/36735	12476.20/28203
	(76.48±1.08%)	(64.18±0.68%)	(8.08±0.55%)	(73.86±1.21%)	(23.38±1.08%)	(44.24±1.22%)

In Table 9, it can be seen that cosine similarity connected more edges than Euclidean distance, implying that it requires more computation. Additionally, more edges are connected between NC and MCI with cosine similarity compared to Euclidean distance. This might explain why GCN-CS-com does not predict NC and MCI as well as GCN-ED-com in Fig 6. While classifying between NC and AD is relatively easy, accurately classifying MCI is more challenging [5, 21, 33, 34]. The GCN-ED-com in this study demonstrated higher accuracy and lower standard deviation than other models in multiclass classification with stratified nested 5 × 4-fold CV, indicating its potential for future applications in AD stage classification.

The key approaches of this study are as follows. First, 3D DenseNet was employed as a feature extractor to obtain PET imaging feature vectors from 3D FBB PET images. These PET imaging feature vectors were then concatenated with non-imaging feature vectors consisting of clinical indicators using a multimodal feature fusion method. This produced combined feature vectors that were used as inputs for multimodal models. Second, various adjacency matrices were constructed using the edge assignment method based on either the cosine similarity or Euclidean distance between the subjects’ PET imaging feature vectors and/or non-imaging feature vectors. In addition, a grid search method was conducted to identify the best modality usage ratio and edge assignment threshold for proper edge assignments. Third, as detailed in Section 2.6, the challenge of identifying the best number of epochs for each deep learning model was addressed by developing a stratified nested 5 × 4-fold CV and incorporating an early stopping method. This nested CV method can prevent overfitting problem and improve the reliability of model evaluation and comparison. Finally, the limitations of the cosine similarity-based edge assignment method in multiclass classification were visually confirmed using PCA and a confusion matrix. These findings were further supported by the numerical edge analysis, as shown in Table 9. We believe that these approaches will contribute to future studies of AD stage classification.

This study has the following limitations. First, we used a relatively simple GNN, GCN, trained with a semi-supervised learning method. Although this approach was effective for our current dataset, it requires full batch learning, which may limit scalability. For larger datasets, a fully supervised GNN will be necessary in future studies. Second, only FBB PET images and numerical clinical indicators were used for population graph construction. We did not use categorical clinical indicators, such as gender and ApoE4, as values were missing for the latter. In future studies, we plan to develop an edge assignment method that can properly incorporate both numerical and categorical clinical indicators. The expected method for including categorical variables in edge assignment can employ one-hot encoding or the Kronecker delta function [14, 32, 37]. Furthermore, by using unsupervised learning methods or neural networks to reduce noisy information [12, 13, 38], a population graph would better represent the relationships between subjects.

## 5 Conclusion

This study demonstrated the effectiveness of GCN for AD stage classification using multimodal data, specifically FBB PET images and clinical indicators from the DAUH and ADNI multimodal datasets. A multimodal feature fusion method was employed to create combined feature vectors using 3D DenseNet. Population graphs were constructed based on either cosine similarity or Euclidean distance between combined feature vectors of subjects. The GCN was compared with SVM, RF, and MLP models using a stratified nested 5 × 4-fold CV to ensure reliable model comparisons.

In the NC vs. MCI vs. AD classification, GCN-ED-com using Euclidean distance-based edge assignment achieved average test accuracies of 90.43% for DAUH and 90.11% for ADNI, outperforming the other models. The GCN-CS-com using cosine similarity-based edge assignment showed relatively lower accuracies of 82.63% for DAUH and 76.16% for ADNI. These performance differences were analyzed visually and numerically in the previous section.

These findings suggest the importance of constructing an appropriate population graph. Future studies are required to develop improved population graph construction methods and employ advanced GNN models to achieve higher accuracy in AD stage classification.

## Supporting information

S1 Table Comparison of average test performances in A*β* positivity classification (mean ± SD). The A*β* is a hallmark of AD indicated by substantial amyloid plaque accumulation in amyloid brain PET images. The A*β* positivity labels were visually determined by a nuclear medicine specialist at DAUH.
(PDF)

S2 Table Average test accuracies of models in NC vs. MCI vs. AD classification robustness tests with Gaussian noise (mean = 0) at varying standard deviations (SD) using the DAUH multimodal dataset. The baseline results correspond to those in Table 8, and Gaussian noise was applied to the standardized non-imaging feature vectors of the test dataset, which were standardized using the training dataset. The models were evaluated using stratified nested 5 × 4-fold CV.
(PDF)

S3 Table Average test accuracies of models in NC vs. MCI vs. AD classification robustness tests with Gaussian noise (mean = 0) at varying standard deviations (SD) using the ADNI multimodal dataset. The baseline results correspond to those in Table 7, and Gaussian noise was applied to the standardized non-imaging feature vectors of the test dataset, which were standardized using the training dataset. The models were evaluated using stratified nested 5 × 4-fold CV.
(PDF)
