# Supplementary material for: Multimodal feature fusion-based graph convolutional networks for Alzheimer’s disease stage classification using F-18 florbetaben brain PET images and clinical indicators
Source: PLoS One. 2024 Dec 23;19(12):e0315809. doi: 10.1371/journal.pone.0315809 (PMC11666044; doi:10.1371/journal.pone.0315809)
Supplement: S1 Table — The Aβ is a hallmark of AD indicated by substantial amyloid plaque accumulation in amyloid brain PET images. The Aβ positivity labels were visually determined by a nuclear medicine specialist at DAUH. (PDF) [file pone.0315809.s001.pdf]

3D CNN Model	Total parameters	Accuracy(%)	Precision(%)	Recall(%)	F1 score(%)	AUC
VGG-16	11,042,850	95.14 ± 1.75	97.56 ± 1.72	94.26 ± 3.46	95.83 ± 1.57	0.9895 ± 0.009
VGG-19	15,026,082	93.91 ± 3.46	97.22 ± 4.04	92.64 ± 4.29	94.77 ± 2.91	0.9812 ± 0.015
ResNet-50	11,563,810	94.66 ± 1.75	97.45 ± 2.13	93.55 ± 2.84	95.42 ± 1.52	0.9836 ± 0.010
ResNet-77	16,738,594	94.12 ± 2.27	96.51 ± 2.64	93.64 ± 3.63	94.98 ± 2.01	0.9845 ± 0.011
DenseNet-46	**1,602,141**	95.73 ± 1.67	97.22 ± 1.77	95.61 ± 2.61	96.38 ± 1.45	**0.9930 ± 0.006**
DenseNet-64	2,580,681	95.78 ± 1.75	**98.16 ± 1.39**	94.71 ± 2.48	96.39 ± 1.53	0.9924 ± 0.006
DenseNet-122	11,754,882	**95.89 ± 1.78**	97.38 ± 1.42	**95.70 ± 2.84**	**96.50 ± 1.55**	0.9903 ± 0.011
EfficientNet-b0	4,690,942	88.42 ± 15.43	88.14 ± 23.21	90.16 ± 20.98	88.58 ± 21.36	0.9171 ± 0.164
EfficientNet-b1	7,449,058	93.59 ± 2.66	95.90 ± 2.24	93.28 ± 3.86	94.52 ± 2.34	0.9823 ± 0.013
EfficientNet-b2	8,717,764	81.83 ± 16.34	83.33 ± 17.56	95.78 ± 3.59	87.73 ± 9.62	0.8115 ± 0.235
EfficientNet-b3	12,061,546	91.08 ± 10.59	92.83 ± 11.18	94.89 ± 3.20	93.31 ± 6.37	0.9399 ± 0.141
EfficientNet-b4	19,616,346	85.82 ± 15.18	87.30 ± 15.99	95.70 ± 3.81	90.19 ± 9.04	0.8674 ± 0.204
