# Supplementary material for: Multimodal feature fusion-based graph convolutional networks for Alzheimer’s disease stage classification using F-18 florbetaben brain PET images and clinical indicators
Source: PLoS One. 2024 Dec 23;19(12):e0315809. doi: 10.1371/journal.pone.0315809 (PMC11666044; doi:10.1371/journal.pone.0315809)
Supplement: S2 Table — The baseline results correspond to those in Table 8, and Gaussian noise was applied to the standardized non-imaging feature vectors of the test dataset, which were standardized using the training dataset. The models were evaluated using stratified nested 5 × 4-fold CV. (PDF) [file pone.0315809.s002.pdf]

**Model**	**Baseline**	**SD=0.1**	**SD=0.2**	**SD=0.3**
SVM-RBF	74.83±5.32	75.04±4.96	74.61±4.54	74.03±4.34
RF	73.01±4.50	73.33±4.49	73.60±4.61	73.60±4.71
MLP-1HL	74.99±4.56	74.89±4.71	74.83±4.87	74.78±5.06
MLP-2HL	76.75±4.85	76.64±4.92	76.54±4.72	76.32±4.70
MLP-3HL	77.66±5.16	77.71±4.79	76.86±4.60	76.33±4.81
GCN-CS-img	71.57±3.47	71.47±3.40	71.36±3.33	71.15±3.67
GCN-CS-nimg	82.26±3.04	81.46±3.17	80.76±2.42	80.45±2.76
GCN-CS-com	82.63±4.01	82.84±3.74	82.09±3.91	81.45±4.16
GCN-ED-img	71.52±2.95	71.63±3.13	71.73±3.21	71.62±3.38
GCN-ED-nimg	89.41±2.25	89.52±2.46	87.76±2.35	84.71±2.51
GCN-ED-com	**90.43±1.78**	**90.59±2.09**	**89.10±2.35**	**86.69±2.39**
