# Supplementary material for: Multimodal feature fusion-based graph convolutional networks for Alzheimer’s disease stage classification using F-18 florbetaben brain PET images and clinical indicators
Source: PLoS One. 2024 Dec 23;19(12):e0315809. doi: 10.1371/journal.pone.0315809 (PMC11666044; doi:10.1371/journal.pone.0315809)
Supplement: S3 Table — The baseline results correspond to those in Table 7, and Gaussian noise was applied to the standardized non-imaging feature vectors of the test dataset, which were standardized using the training dataset. The models were evaluated using stratified nested 5 × 4-fold CV. (PDF) [file pone.0315809.s003.pdf]

**Model**	**Baseline**	**SD=0.1**	**SD=0.2**	**SD=0.3**
SVM-RBF	79.08±8.04	76.29±9.62	69.44±10.34	66.45±7.56
RF	77.43±12.22	76.04±11.37	72.95±11.29	70.11±12.08
MLP-1HL	74.60±9.88	73.46±10.33	73.43±9.78	72.30±10.01
MLP-2HL	75.37±8.59	74.80±8.82	74.49±8.71	76.60±7.41
MLP-3HL	74.28±8.92	73.12±8.05	72.56±9.30	70.27±9.55
GCN-CS-img	72.05±10.34	71.76±10.84	71.45±10.89	69.18±12.61
GCN-CS-nimg	75.32±5.54	73.26±5.48	73.49±7.03	74.06±7.31
GCN-CS-com	76.16±6.75	76.07±7.61	74.39±8.01	74.67±10.08
GCN-ED-img	64.33±14.01	62.59±14.26	61.45±13.67	60.86±13.05
GCN-ED-nimg	85.81±9.92	82.71±8.97	78.51±10.71	75.35±10.01
GCN-ED-com	**90.11±5.28**	**87.85±5.84**	**82.66±6.19**	**78.15±6.75**
